# Ameliorative effects of crocin against electromagnetic field-induced oxidative stress and liver and kidney injuries in mice

**DOI:** 10.22038/AJP.2022.21169

**Published:** 2023

**Authors:** Azam Vafaei, Ahmad Reza Raji, Mohsen Maleki, Mahdieh Zaeemi, Alireza Ebrahimzadeh-bideskan

**Affiliations:** 1 *Faculty of Veterinary Medicine, Ferdowsi University of Mashhad, Mashhad, Iran*; 2 *Microanatomy Research Center, Mashhad University of Medical Sciences, Mashhad, Iran*

**Keywords:** Crocin, Electromagnetic field, Oxidative stress, Liver and kidney, Mice

## Abstract

**Objective:**

The current study's goal was to examine how crocin affects organ damage such as damage to the kidney and liver in mice treated by 2100 MHz Electro Magnetic Field.

**Materials and Methods:**

The liver and kidneys of mice exposed to EMFs were used in this study to examine how crocin affected them. 24 male NMARI mice were randomly divided into 4 groups: EMF group (2100 MHZ); Crocin (Cr) group (50 mg/kg); EMF+Crocin group (2100 MHZ+50 mg/kg), and control group. The antioxidant enzymes and some serum biochemical parameters were assessed in blood samples collected after the experiment. After the animals were put to sleep, liver and kidney samples were taken for histopathological and liver samples were taken for ultrastructural analyses.

**Results:**

The serum levels of urea and creatinine, and serum activities of alanine transaminase, aspartate transaminase, and alkaline phosphatase were higher in the EMF group than the control group, and this difference was significant. When compared to the control group, the EMF group's antioxidants, (catalase and superoxide dismutase) activity were decreased. These metrics significantly improved in the EMF + Cr group when compared to the EMF group. Different pathological damages were present in the liver and kidney of the EMF group, and the liver's ultrastructure had changed. Crocin administration decrease these changes.

**Conclusion:**

Crocin, an antioxidant agent, may provide defense against tissue damage brought on by EMF by reducing oxidative stress.

## Introduction

People are inevitably exposed to ambient electromagnetic fields (EMF), which are produced by various power transmission lines and electrical devices. Without realizing it, every piece of electronic equipment we use daily emits electromagnetic fields (EMF) (Ongel et al., 2009). Extremely Low-Frequency Electromagnetic Fields (ELF-EMF), in particular, have permeated modern life as a result of the expanding use of electric technology. All electric devices generate these fields, including high-energy sources like power lines and microwaves and low-energy ones like mobile phones (Van Deventer et al., 2005).

A cellular phone is defined as a gadget that emits radiofrequency electromagnetic waves (RF-EMW). These waves deliver cell phone signals to the antennas and base stations. The frequency of these waves is low, ranging from 800-2200 MHz. Human users are still at risk because their bodies can act as antennas to absorb these waves and convert them into eddy currents (Andersen et al., 2002; Barnett et al., 2007).

Concern has been raised about potential side effects on living things due to the recent increase in cell phone use and increased exposure to EMF. However, most people are unaware of how these devices' electro magnetic field emissions can harm their health (Elbetieha et al., 2002).

Due to the proximity of this device's antenna to the abdominal organs when it is fastened to a belt, worries have been raised about the kidney and liver as well as the biological interactions between electromagnetic radiation (EMR) (Ragy, 2015; Kumar, 2012).

Free radical production by mobile phones has reportedly been observed in other tissues (Irmak et al., 2002). Researchers discovered that electromagnetic fields increase the free radical activity of cells (Grundler et al., 1992). According to *in vivo* studies, cell phone radiation causes oxidative stress (OS) in animals (Balci et al., 2007; Ozguner et al., 2006). RF-EMW have the potential to alter metabolism by boosting the generation of reactive oxygen species (ROS) or lowering the activity of antioxidant enzymes. As a result of repeated exposure to RF-EMW, superoxide dismutase (SOD) and catalase (CAT) activities decrease thus, decreasing the total antioxidant capacity (Balci et al., 2007; Dasdag et al., 1999; Oral et al., 2006).

Numerous studies have shown that ROS are directly responsible for oxidative damage to cellular macromolecules in tissues, including lipids, proteins, and nucleic acids (Ilhan et al., 2004; Iramk et al., 2003). ROS have been related to tissue damage (Ozguner et al., 2005). Some theories contend that ROS and free radicals contribute to the biological effects of EMFs (Moustafa et al., 2001).

EMFs have an impact on a number of cellular processes, according to numerous studies on animal cells (Lagroye and Poncy, 1998). One or more of the reported mechanisms of interaction with living cells include changes in intracellular Ca^2+^ levels (Lyle et al., 1997). However, several proposed theories assume that the initial field encounter will most likely target the cell membrane, and this interaction may have varying degrees of influence on signal transduction mechanisms (Bersani et al., 1997).

Adaptive modifications, which alter lactate dehydrogenase activity and speed up transamination processes, are likely brought on by the disturbances. EMFs enter the body of a person and have an impact on all organs, altering the distribution of ions and dipoles as well as the potential of cell membranes. These changes may impact cellular biochemical processes, altering serum enzyme activities and biochemical parameters (Duda et al., 1991). Antioxidant therapies for humans and animals may help prevent or lessen some side effects of low-frequency EMF. 

In developing nations, phytomedicine has recently gained popularity as an alternative treatment for various health-related issues (Jin et al., 2013). Due to their redox properties, different plant extracts and their constituents, including phenolic compounds and flavonoids (Ebrahimzadeh et al., 2010), have antioxidant activity and can bind free radicals (Padma et al., 2011). The most prevalent antioxidant compound in *Crocus sativus* L stigma, crocin, a water-soluble carotenoid, gives saffron its distinctive red color (Jalili et al., 2015). It has been proven that crocin has antiapoptotic, anti-inflammatory, and antioxidant properties (Mard et al., 2016). Additionally, crocin has been shown to have well-established retino- (Qi et al., 2013), gastro- (Mard et al., 2015) protective effects against ionizing radiation -induced injury.

The current study's goal was to examine how crocin affects organ damage such as damage to the kidney and liver in mice treated by 2100 MHz EMF.

## Materials and Methods

**Animals**

For this study, the Mashhad University of Medical Sciences, Iran, Animal Center provided 24 male NMARI mice that were 60 days old and weighed 30–40 g. The animals were kept in typical laboratory conditions throughout the experiment, including an air-conditioned room with a constant humidity of between 40 and 50 percent, a 12/12 hr light/dark cycle, free access to food and water (Jalili et al., 2014).

**Crocin preparation**

Crocin powder was offered for sale by the pharmacy school at Mashhad University of Medical Sciences. It was acquired and kept in a cool, dry location. For intraperitoneal injection, crocin was dissolved in distilled water at a 10 mg/ml concentration every day. To account for the various mouse weights, the injection volume (50 mg/kg) was changed (Hosseinzadeh et al., 2005).

**Electromagnetic field (EMF)**

**Figure 1:**
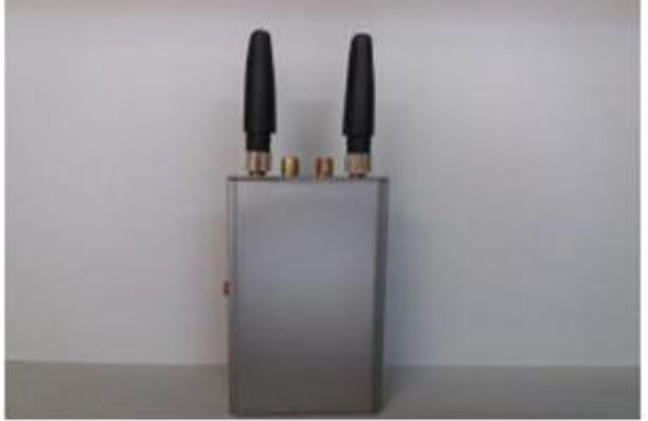
Electromagnetic field-producing device (2100 MHz)

In the current study, the electromagnetic field was generated using a device (Figure 1) that produces a 2100 MHz Electromagnetic field, which is comparable to that of 4G-LTE mobile phones.

**Experimental design**

Four groups, including 24 mice, were created randomly: 

1) For 30 consecutive days, the animals in the EMF group were given injections of distilled water (1 ml per 100 g body weight) while being exposed to 2100 MHz of EMF for two hours each day. 

2) For 30 days, the animals in the EMF+Crocin group were given injections of crocin (50 mg/kg/day) while being exposed to 2100 MHz of EMF for two hours each day (Abdullaev, 1993; Ebrahimzadeh et al., 2010). 

3) The Crocin (Cr) group's animals received crocin injections (50 mg/kg/day) for 30 consecutive days (Abdullaev, 1993; Ebrahimzadeh et al., 2010). 

4) Control group without any intervation

Blood samples were taken after the experimentation was completed, and the animals' livers and kidneys were removed.

**Biochemical assay**

Serum samples were prepareded by centrifuging of the blood at 1500 g for 10 min, and they were then kept at 80°C pending analysis. The serum levels of urea, creatinine, aspartate aminotransferase (AST), alkaline phosphatase (ALP), and alanine aminotransferase (ALT) were measured using an auto-analyzer (Mindray, China) and commercial kits (Pars Azmoon, Tehran, Iran).

Commercial kits (Navand Salamat, Urmia, Iran) were used in accordance with the manufacturer's instructions to measure the serum levels of catalase and superoxide dismutase. Enzyme activity is expressed in units per milliliter (U/ml).

**Histopathological analysis**

The kidney and liver samples were prepared for histological analysis by being fixed in 10% formalin, sectioned into 5 µm pieces using a microtome, and mounted on glass slides. The prepared slides were examined under a light microscope, stained using the Hematoxylin-Eosin technique, and captured on camera for analysis.

**Electron microscopy**

Small (1 mm) pieces of liver were cut out for electron microscopy examination and immediately fixed in 2-4% cold phosphate-buffered glutaraldehyde. The samples were thoroughly cleaned at 4°C in 0.1 N of the buffer solution after fixation. After which, they were dehydrated and embedded in epoxy resin after being post-fixed for two hours in 1% osmium tetraoxide in phosphate buffer. After being stained with lead citrate and alcoholic uranyl acetate, extremely thin sections were examined under a transmission electron microscope.

**Statistical analysis**

Utilizing SPSS software, the statistical analysis was performed. By means of the Kolmogorov-Smirnov test, the distribution of the measured variables was evaluated. Using the *post hoc* Bonferroni test, two groups were compared after using the one-way ANOVA test for intergroup comparisons. Values are expressed using the standard error of the mean (SEM), and statistical significance was set at p<0.05.

## Results

**Effects of crocin treatment on the serum activity of liver enzymes after exposure to electromagnetic fields (EMF)**

**Figure 2:**
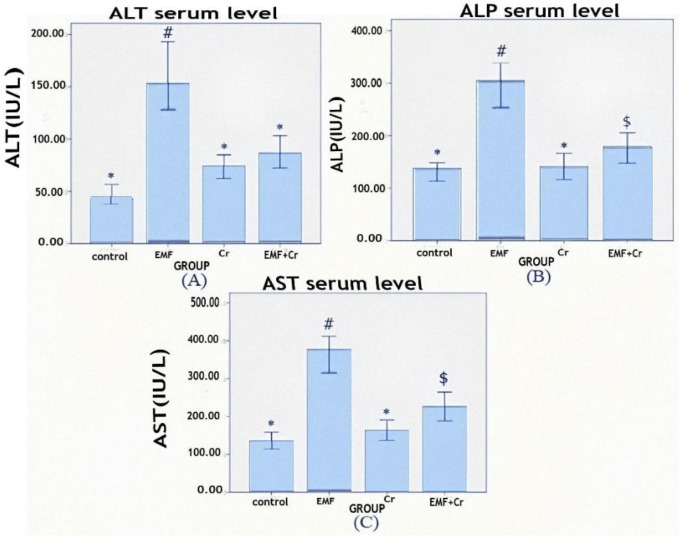
Comparison of liver enzyme serum activities (mean±SEM) between different groups (control, EMF: electromagnet field, Cr: Crocin, and EMF+Cr: electromagnet field+Crocin). A) ALT: Alanine aminotransferase, B) AST: Aspartate aminotransferase, and C) ALP: alkaline phosphatase. Dissimilar symbol showed a significant difference (*p<0.05, #p<0.05, $p<0.05).

Comparing the EMF group to the control group, the serum activity of liver enzymes were significantly higher after the EMF. Even though this research showed that crocin treatment significantly decreased all liver enzyme activities in the EMF+Cr group, the serum activity of AST and ALP was still significantly higher (p<0.05) than that of the control group (Figures 2A, 2B, and 2C).

**Crocin's impacts on serum levels of urea and creatinine after exposure to electromagnetic fields (EMF)**

**Figure 3:**
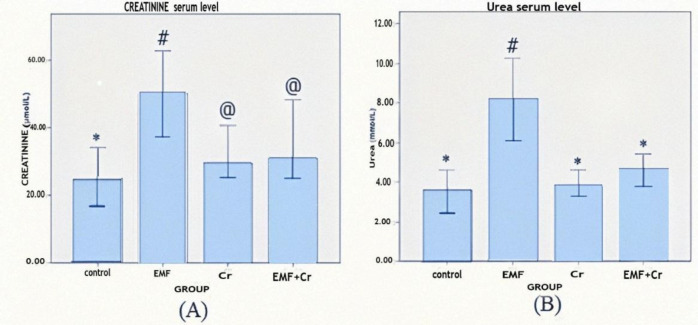
Comparison of serum level (mean±SEM) of creatinine (A) and urea (B) between different groups (control, EMF: electromagnet field, Cr: Crocin, and EMF+Cr: electromagnet field + Crocin). Dissimilar symbol showed a significant difference (*p<0.05, #p<0.05, @p<0.05).

When compared to the control group, the serum concentrations of urea and creatinine were significantly higher in the EMF group (p<0.05). Despite reducing serum urea and creatinine levels, crocin could only significantly (p<0.05) lower urea concentration in the EMF+Cr (Figure 3A and 3B). 

**Effects of crocin treatment on serum SOD and CAT activity after electromagnetic field exposure (EMF)**

**Figure 4:**
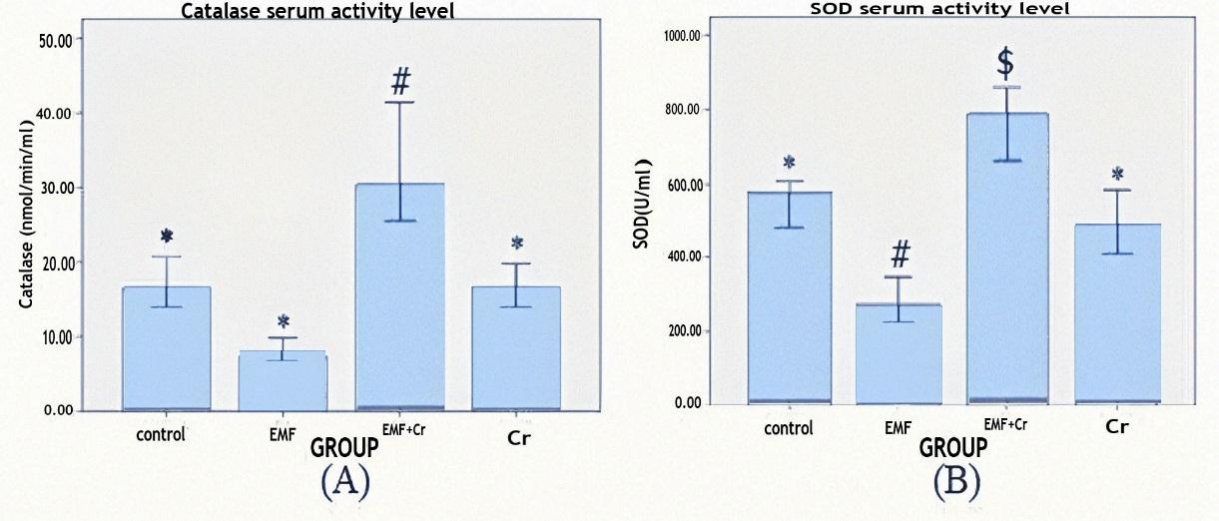
Comparison of serum activities (mean±SEM) of catalase (A) and superoxide dismutase (B) enzymes between different groups (control, EMF: electromagnet field, Cr: Crocin, and EMF+Cr: electromagnet field + Crocin). Dissimilar symbol showed a significant difference (*p<0.05, #p<0.05, $p<0.05).

When compared to the control group, the serum activity of SOD in the EMF group decreased significantly (p<0.05, Figure 4A). Although serum CAT activity decreased in the EMF group, this change was insignificant (Figure 4B). Crocin treatment led to increment in the activity of both enzymes but in SOD was significant (p<0.05). 

**Effects of crocin treatment on histopathological changes in kidney and liver tissue following exposure to electromagnetic fields (EMF)**

**Kidney**

**Figure 5:**
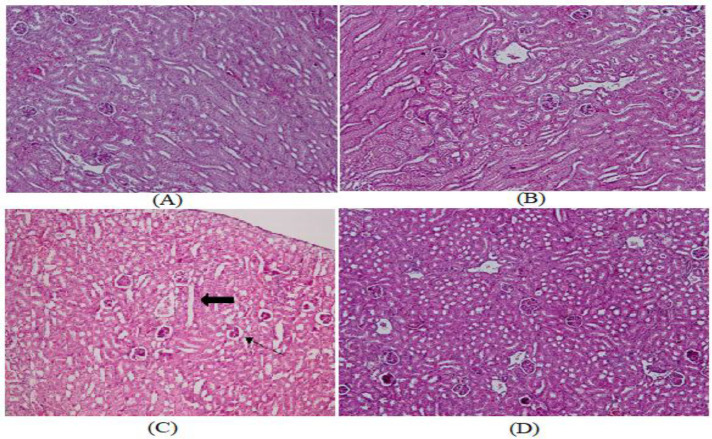
Hematoxylin-Eosin staining (100x magnification) of kidney histological changes. A. Micrograph of a mouse kidney section from the control group demonstrates a normal kidney structure. B. A normal kidney structure is visible in the micrograph of a kidney section after treatment with Cr (50 mg/kg). C. An enlarged Bowman's space, atrophied glomeruli, and deteriorated renal tubules can be seen in a micrograph of a kidney section exposed to EMF (thick arrow). D. A micrograph of the kidney section of the EMF+Cr group demonstrating improved kidney structure.

Hematoxylin and eosin-stained kidney tissue samples from the control and Cr groups revealed normal liver structure. After 30 days of EMF exposure, the kidney section showed various changes and an obvious injury. Compared to the control group, these changes manifested as Bowman's space enlargement, atrophied glomeruli, clogged blood vessels, and degenerated renal tubules. It was discovered that crocin (50 mg/kg) treatment for 30 days reduced kidney damage brought on by EMF (Figure 5).

**Liver**

**Figure 6:**
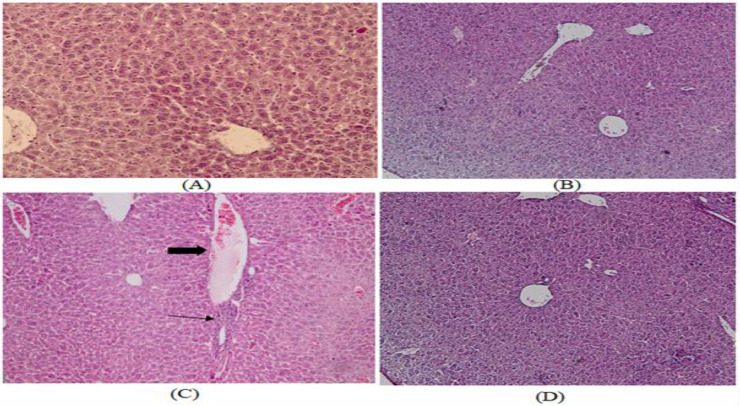
Liver histological modifications (100 times) Hematoxylin-Eosin staining. A. Microscopic image of a mouse liver section from the control group demonstrating a typical liver structure. B. A normal liver structure can be seen in the micrograph of a liver section after treatment with Cr (50 mg/kg). C. A micrograph of a liver section exposed to EMF reveals an enlarged central hepatic vein (thick arrow) and more lymphocytes infiltrating the area around it (thin arrow) D. Micrograph of the EMF +Cr group's liver section demonstrating the normal liver structure.

By looking at the hematoxylin and eosin-stained liver sections in the control and Cr groups, the normal liver structure was demonstrated. After 30 days of exposure to EMF, the liver section displayed a variety of modifications and clear damage. These changes could be seen as a disruption in tissue architecture, an increase in lymphocyte infiltration around the central vein, and an enlargement of the central hepatic vein when compared to the control group. After 30 days of treatment, it was found that crocin (50 mg/kg) decreased the liver damage caused by EMF (Figure 6). 

**Effects of crocin treatment on ultrastructure changes in the liver following exposure to electromagnetic fields (EMF)**

**Figure 7:**
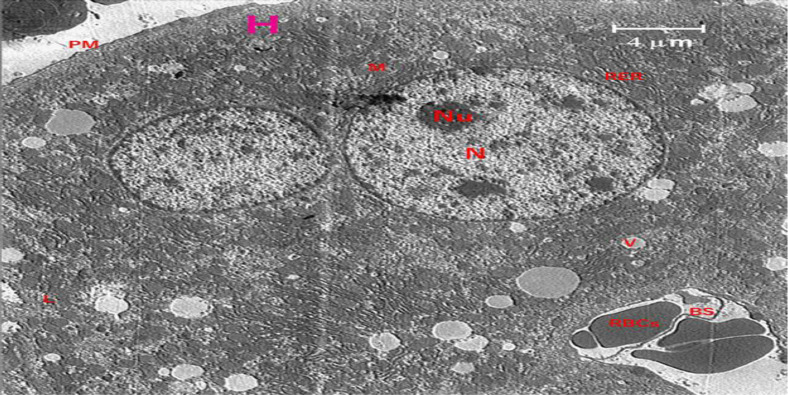
The electron micrograph of a control liver shows lysosomes (L), rough endoplasmic reticulum (RER), scattered mitochondria (M), double intact nuclei (N), double intact nucleoli (Nu), and hepatocytes (H) (L). A blood sinusoid (BS), and red blood cells (RBC).

**Figure 8:**
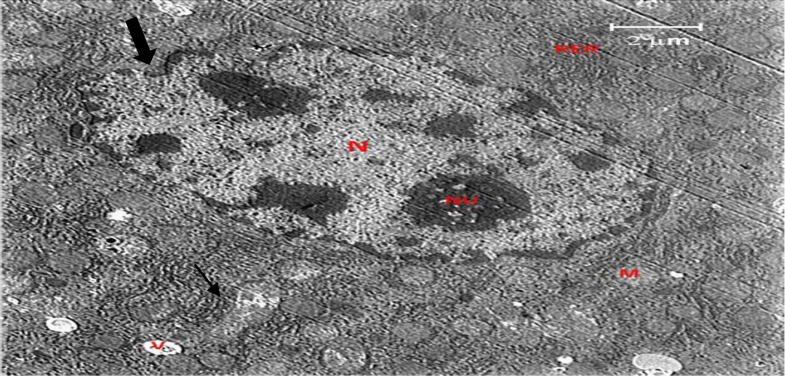
A hepatocyte with an indented nucleus (thick arrow) and some clumped heterochromatin, fragmented rough endoplasmic reticulum (RER), and swollen mitochondria (thin arrow) (2µm) is shown in an electron micrograph of the liver from the EMF group.

A section of the liver from the control group is shown in Figure 7. The findings demonstrated that structural disorder in hepatocyte nuclei was the most obvious ultrastructural alteration in the liver of the EMF group (Figure 8). Increasing nuclear pores, irregular nuclear membranes, and a heterogeneous distribution of the chromatin material were the results.

**Figure 9:**
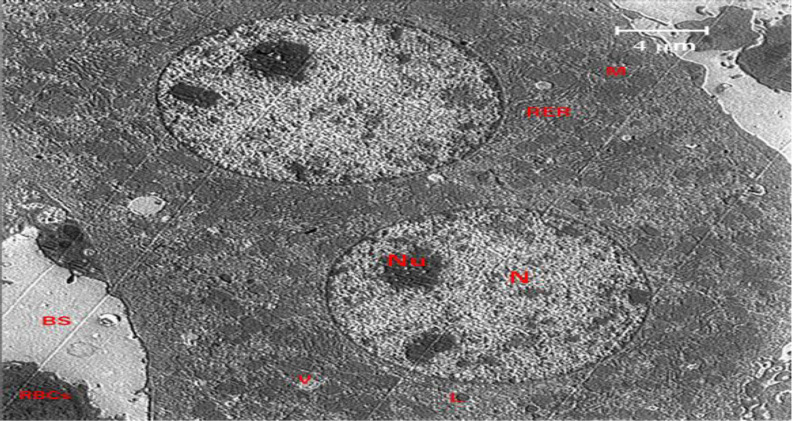
Electron micrograph of the EMF +Cr group liver showing a hepatocyte with reduced injury

Additionally, the groups exposed to the magnetic field showed clumping, swelling, and deformation of the mitochondria. The rough endoplasmic reticulum displayed prominent dilation, and the lysosomes appeared distorted. It was discovered that taking crocin (50 mg/kg) for 30 days reduced the liver damage caused by EMF (Figure 9).

## Discussion

In the current study, mice exposed to EMR at a frequency of 2100 MHz showed protective effects of crocin on their kidney and liver. Concerns have been raised by scientists regarding the potential risks posed by recently developed technology, such as mobile phones. The primary sources of EMF emissions are mobile phones, and their use is expanding globally (Narayanan et al., 2010). In the current study, the biochemical parameters of the liver, kidneys, and oxidative stress in mice exposed to EMR were evaluated.

The data collected showed that EMR significantly altered liver enzymes like ALT, AST, and ALP, which are frequently used as biomarkers of specific organ dysfunction in mammalian toxicology. Increased transaminase activity is typically associated with hepatocyte damage (Sihem et al., 2006).

A new membrane conformation may result from significant structural alterations in the protein molecules embedded in the cell membrane due to exposure to a time-varying magnetic field, which induces EMF. In this new conformation, the protein molecule forms a temporary bond with the ions, allowing the ions to "hop" through the membrane (Valberg Alberg et al., 1997). The distribution of ions and dipoles in the cell membrane as well as the serum levels of ALT and AST, two indicators of hepatotoxicity, may change as a result (Watanabe et al., 1997).

The present research findings indicated that the EMF+CR group's liver enzymes responded favorably to crocin treatment. According to reports, crocin decreased the amount of these enzymes after nicotine-caused hepatic injury (Jalili et al., 2015).

Information on the condition of the kidneys' health has been gleaned from estimates of waste metabolite excretion in the kidneys and renal tissue's histological changes. Due to protein metabolism, the chemical waste products creatinine and urea are transported via the bloodstream to the kidneys and excreted in the urine (Panda, 1999). The current findings demonstrate that exposure to EMF enhances the serum contents of creatinine and urea. Cell phone radiation and cellular damage have been linked, according to Grundler et al. (1992), which could lead to higher serum urea and creatinine concentrations.

The results demonstrated the effects of crocin treatment on urea and creatinine. However, the serum creatinine level between the EM and EMF+Cr did not significantly differ. It appears that longer treatment periods or higher doses may be required to achieve the positive effects of crocin on renal function. 

Oxidative radiation from radiation activates the apoptotic pathway (Ozben, 2007). Increased ROS concentrations within cells or physiological systems cause oxidative stress, which results in molecular harm to important functions and structures (Yasser et al., 2001). According to reports, radiation damages cell membranes and causes lipid peroxidation, which sends an apoptotic signal (Ozben, 2007). Radiation may mediate its apoptotic effects by causing an increase in oxidative stress (Ozguner et al., 2005). Radiation from mobile phones has increased the formation of free radicals in other tissues (Ilhan et al., 2004; Irmak et al., 2002).

SOD and CAT scavenge these continuously produced ROS. Due to excess oxygen radical production, deactivation of detoxification systems, antioxidant consumption, and inadequate antioxidant replenishment in tissue may occasionally compromise these endogenous antioxidant defenses (Ozguner et al., 2005).

SOD and CAT activity, however, significantly decreased in serum. The findings of our study supported the idea that EMF might result in oxidative injury to liver tissue. The liver's lipid peroxidation reactions may be accelerated by oxidative stress (Luo et al., 2016).

In the present research, we demonstrated that after exposure to EMF, the serum activity of SOD and CAT decreased. According to a study, CAT and SOD mRNA expression reduced after oxidative stress (Rajaei et al., 2013).

Crocin has the ability to reduce the amount of ROS produced by various oxidative stresses (Ochiai et al., 2004). The carotenoids found in saffron extracts may prevent oxidative damage to tissues because of their antioxidant effect. Data demonstrate that crocin alters oxidative markers in the liver, brain, and kidney in a striking manner (Zheng et al., 2007).

The present results also showed that crocin treatment increased the activity of antioxidant enzymes (CAT and SOD) that were tested. The current study found that mice exposed to EMF had enlarged Bowman's spaces, atrophied glomeruli, clogged blood vessels, and degenerated renal tubules in their kidneys.

According to some research findings, renal impairment in animals exposed to mobile phone radiation is caused by oxidative stress brought on by EMFs, and melatonin (an antioxidant) use can protect against this impairment (Hanafy et al., 2010; Oktem et al., 2005; 52). Different oxidative stresses can generate ROS, which can be suppressed by crocin (Ochiai et al., 2004). The kidney damage brought on by exposure to EMF appears to be lessened by antioxidants, such as crocin.

Mice exposed to EMF in the present study showed altered tissue architecture, enlarged central hepatic veins, and lymphocyte infiltration around them. Some researchers claim that EMF causes histopathological changes in the liver, such as necrosis, sinusoidal dilation, vacuolar degeneration, and inflammatory cell infiltration (Erpek et al., 2007; Gocimen et al., 2002). In the present research, Mice exposed to EMFs only had mononuclear cell infiltrations in their livers, and it was found that the amount of inflammatory cell infiltration increased throughout the exposure. The unknown is the mechanism by which EMFs harm tissues and cells. EMFs, nevertheless, have reportedly been linked to damage because they change cell membrane proteins' electrical structure and raise free radical levels (Seyhan and Canseven, 2006; Tsong, 1992). Additionally, researchers found that the cytochrome P-450 enzyme system and free oxygen radicals induced by EMF in the liver resulted in lipid peroxidation of plasma and organelle membranes and hepatic injury (Koyu et al., 2009).

Crocin's antioxidant properties could have caused the decrease in lymphocyte activation. It is hypothesized that crocin's anti-inflammatory properties primarily account for its ability to lower Tumor necrosis factor alpha, Interleukin-1 and ROS levels (Nam et al., 2010). Crocin appears to have lessened the negative impacts of EMF exposure on the liver because of its antioxidant properties.

When exposed to alternating current EMFs, the nucleus of the hepatocyte from animals was indented in the current study. Furthermore, mitochondrial swelling, clumping, and deformation were seen in the groups exposed to the magnetic field. Additional indications of cell damage include fragmentation of the rough endoplasmic reticulum. These results are consistent with earlier research (El-Hady El-Desoky and Mohamady, 2011).

Reports state that the effects of EMF on cellular activity are due to structural and functional changes in cell membranes that lead to the intracellular mobilization of intracellular calcium ions (Tonini et al., 2001). The cause of changes in cell structure and function has also been proposed to be free radicals released in tissues and cells exposed to EMF (Forgács et al., 2004). Free radicals and ROS alter the structure and function of the mitochondria and nucleus (Kergonou et al., 1981). The vascularization, dilatation, degranulation, and fragmentation of the endoplasmic reticulum cisternae were all effects of excessive free radical release in different cells (Skog et al., 1983).

Through a decrease in biochemical factors, an increase in antioxidant activity, and an improvement in histopathological changes in tissue, this study demonstrated that crocin treatment has protective effects after exposure to EMF. As a result, this organic compound may be beneficial for kidney and liver conditions.

## Conflicts of interest

The authors have declared that there is no conflict of interest.
